# *Escherichia coli* O157 : H7 from Food of Animal Origin in Arsi: Occurrence at Catering Establishments and Antimicrobial Susceptibility Profile

**DOI:** 10.1155/2021/6631860

**Published:** 2021-03-29

**Authors:** Minda Asfaw Geresu, Shimelis Regassa

**Affiliations:** ^1^Department of Veterinary Science, College of Agriculture and Environmental Science, Arsi University, Asella, Ethiopia; ^2^Department of Animal Science, College of Agriculture and Environmental Science, Arsi University, Asella, Ethiopia

## Abstract

*Escherichia coli* O157 : H7 (*E. coli* O157 : H7) has been found to be the major cause of food-borne diseases and a serious public health problem in the world, with an increasing concern for the emergence and spread of antimicrobial-resistant strains. Hitherto, little is known about the carriage of *E. coli* O157 : H7 and its antimicrobial susceptibility profile in the food of animal origin in Ethiopia. This study aimed to determine the occurrence and multidrug resistance profile of *E. coli* O157 : H7 from food of animal origin at different catering establishments in the selected study settings of Arsi Zone. One hundred ninety-two animal origin food items, namely, raw/minced meat (locally known as “Kitfo,” “Kurt,” and “Dulet”), raw milk, egg sandwich, and cream cake samples were collected and processed for microbiological detection of *E. coli* O157 : H7. Out of 192 samples, 2.1% (4/192) were positive for *E. coli* O157 : H7. Two *E. coli* O157 : H7 isolates were obtained from “Dulet” (6.3%) followed by “Kurt” (3.1%, 1/32) and raw milk (3.1%, 1/32), whereas no isolate was obtained from “Kitfo,” egg sandwich, and cream cake samples. Of the 4 *E. coli* O157 : H7 isolates subjected to 10 panels of antimicrobial discs, 3 (75%) were highly resistant to kanamycin, streptomycin, and nitrofurantoin. Besides, all the isolates displayed multidrug resistance phenotypes, 3 to 5 antimicrobial resistance, amid kanamycin, streptomycin, nitrofurantoin, tetracycline, and chloramphenicol. The occurrence of multidrug-resistant *E. coli* O157 : H7 isolates from foods of animal origin sampled from different catering establishments reveals that the general sanitary condition of the catering establishments, utensils used, and personnel hygienic practices did not comply with the recommended standards. Thus, this finding calls for urgent attention toward appropriate controls and good hygienic practices in different catering establishments dealing with consuming raw/undercooked foods of animal origin.

## 1. Introduction

Food-borne pathogens are the leading causes of food-borne human illness and death in the world [1]. The severity is higher among developing countries, including Ethiopia [2, 3]. This could be attributed to changes in eating habits, mass catering, complex and lengthy food supply procedures with increased international movement, poor food handling and sanitation practices, inadequate food safety laws, weak regulatory systems, lack of financial resources, and awareness about proper food handling which creates a conducive environment for the spread of food-borne and food poisoning etiologic agents [4–6]. A wide range of pathogens plays a role in food-borne disease, most of which have a zoonotic origin and have carriers in healthy food animals from which they spread to an increasing variety of foods of animal origin and are considered as major vehicles of food-borne infections [7, 8]. Among the bacterial pathogens, *Escherichia coli* O157 : H7 (*E. coli* O157 : H7) has frequently been associated with food-borne illness [9]. The infection caused by these bacteria initially causes nonbleeding diarrhea accompanied by abdominal cramps. Then, it may develop into bloody diarrhea and hemolytic uremic syndrome (HUS), which causes kidney failure in humans [10–12].

Enterohemorrhagi*c E. coli* O157 belongs to the larger category of Shiga toxin-producing *E. coli* (STEC), which can produce Shiga toxin type 1 (Stx1), Shiga toxin type 2 (Stx2), or both, along with other variants [13–15].

*E. coli* O157 can infect humans via various routes; however, a large proportion of infections and human outbreaks have occurred following the consumption of contaminated food products of animal origin, such as raw milk, uncooked or poorly cooked meat (especially in Ethiopia, most people prefer to eat raw or undercooked beef, locally called “Kitfo,” “Dulet,” and “Kurt”) [3], cream, creamed fish, vegetables, and poultry and their products which are generally regarded as a high-risk commodity in respect of pathogen contents, natural toxins, and other possible contaminants and adulterants [16–19].

Antibiotic use in *E. coli* O157 : H7 (STEC O157) infections is controversial because of the potential to increase production and secretion of Shiga toxins, thus promoting the onset of HUS in humans [20]. However, early administration of antibiotics such as rifaximin, fosfomycin, azithromycin, and meropenem was found to not stimulate the release of Shiga toxin from O157 and non-O157 strains in vitro [21]. These antibiotics have been recommended for the treatment of early stages of STEC disease to prevent HUS [22].

Unfortunately, inappropriate ways of antimicrobial uses have contributed to the increase in antimicrobial resistance [3, 23]. Antibiotic resistance in *E. coli* O157 : H7 has been increasingly noted over the last 20 years [24]. A recent study revealed that a higher incidence rate of *E. coli* O157 : H7 multidrug resistance, to more than two antimicrobial agents, was observed in Ethiopia [9, 25–27].

In Ethiopia, a country which ranks second after Nigeria in the health burden of zoonotic diseases in Africa [28], the epidemiology of food-borne pathogens especially that of pathogenic *E. coli* is not well studied. However, recently, there is an increasing trend of reporting the occurrence level of the organism in beef, dairy products, and fish [2, 3, 9, 16, 29–37]. Besides this, current and detailed studies on the prevalence of antibiotic resistance and multidrug phenotypes of enterohemorrhagi*c E. coli* O157 : H7 in foods of animal origin in different catering establishments are very limited in Ethiopia except for the report of [2, 3, 9] on beef at butcher shops and restaurants and raw milk and dairy products such as yogurt and cheese derived from raw milk at cafeterias, restaurants, open markets, and supermarkets in Central Ethiopia.

Though scarce studies revealed that, as *E. coli* O157 is the leading food-borne pathogens that seriously devastate the economic growth of the country, there is a paucity of information (no published data) about the occurrence and multidrug resistance profile of *E. coli* O157 : H7 in animal origin food items in different catering establishments in Arsi Zone in general and in the selected study settings and its suburbs in particular. Hence, this study was conducted to determine the occurrence and multidrug resistance profile of *E. coli* O157 : H7 in the food of animal origin at different catering establishments in the selected study settings of Arsi Zone so as to complement the paucity of information associated with *E. coli* O157 food-borne infections that potentially affect the economic growth of a country and to create awareness in the public and formulate preventive measures along with food production, processing, and distribution continuum in the area.

## 2. Materials and Methods

### 2.1. Study Area

**Figure 1:**
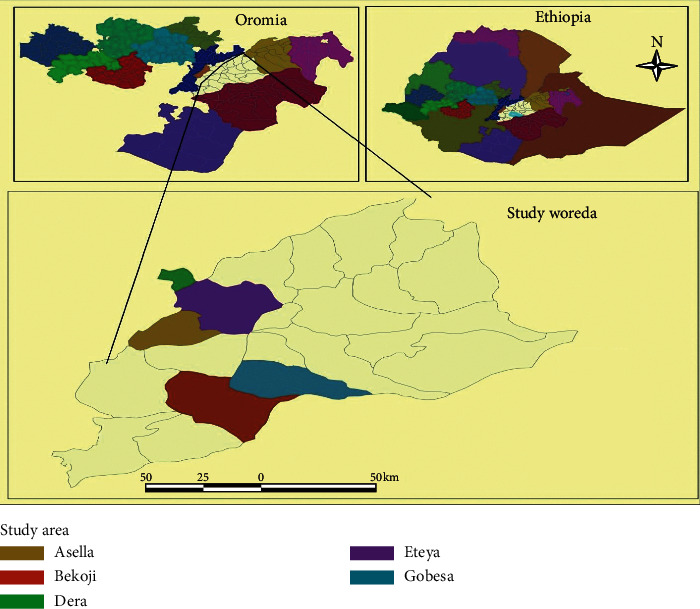
A map that shows the study areas.

The study was conducted in different catering establishments of Arsi Zone of Oromia Regional State, southeastern Ethiopia, from October 2018 to May 2019. Arsi is one of the zones in the Oromia Regional State of Ethiopia which has a population of about 3.5 million. It is located at 6°45′N to 8°58′N and 38°32′ E to 40°50′ E in Central Ethiopia. Five towns from Arsi Zone, namely, Dera [38], Eteya [39], Asella [40], Bekoji [41], and Gobessa, [42] were included in this study (Figure 1).

### 2.2. Study Design

A cross-sectional study was conducted from October 2018 to May 2019 to isolate, identify, and characterize multiple drug resistance *Escherichia coli* O157 : H7 isolates from selected food of animal origin in selected towns of Arsi Zone and its suburbs.

Stratified random sampling from catering establishments and a list of frames of public places were used as a sample source according to the accessibility of animal origin food items. First, functional catering establishments registered in the towns were searched and then public places were stratified into strata (hotels, restaurants, cafeterias, and retail shops) and they were used as a sampling frame. The studied foods of animal origin were collected during dining time using a simple random sampling method in each public place on a proportional basis/considering the availability of the food items.

### 2.3. Sample Size and Study Methodology

Selected food of animal origin samples was purchased from hotels, restaurants, and cafeterias including minced meat (locally known as “Kitfo,” “Dulet,” and “Kurt”), egg sandwich, and cream cake whereas raw milk was bought from retail shops/cafeteria. The time of sampling was scheduled at the beginning of the serving period (breakfast or lunchtime).

The sample size calculation was based on a 50% prevalence assumption (since there was no study on *E. coli* O157 : H7 from different food of animal origin in the selected towns of Arsi Zone), 95% CI, and d*f* = 0.05 [43]. Though the sample size calculated for *E. coli* O157 : H7 isolate was 384, only 192 samples were processed for bacteriological detection of *E. coli* O157 : H7 isolates because of the limited number of catering establishments in the study settings as per our stratification. Hence, the total sample size was divided for the six (6) foods of animal origin that were sampled (viz. 32 samples for each animal origin food were considered to maintain proportionality).

#### 2.3.1. Method of Sample Collection

The selected foods of animal origin including minced meat, egg sandwich, and cream cake samples purchased from the catering establishments were put in sterile plastic bags by using sterile forceps and spoons from the eating plate whereas approximately 10 ml of raw milk bought from different cafeteria/retail shops was collected by using sterile screw capped bottle. All the collected samples were properly identified by sample type, date of collection, and sources and immediately transported to the laboratory (Asella Regional Laboratory, Veterinary Microbiology Section) in an icebox with freeze packs under completely sterile conditions for microbiological analysis according to [44].

#### 2.3.2. Isolation and Identification of *E. coli* O157 : H7

Isolation and identification of *E. coli* O157 : H7 were performed by standard bacteriological methods. Ninety ml of modified tryptone soy broth (mTSB) supplemented with novobiocin (mTSB + N; Oxoid) was added to 10 ml swab sample. Conversely, 25 g of meat (minced), egg sandwich, and cream cake sample was collected in a Stomacher bag. After adding 225 ml mTSB + N, each sample was homogenized using a Stomacher 400 (Seward Medical, England) for two minutes and transferred into a sterile flask. Then, the samples were incubated at 37°C for 24 hours on the Mac Conkey agar (Oxoid Ltd., Cambridge, UK) which is a selective and differential medium for *E. coli* [45]. Then, a pink colony was picked and subcultured onto Eosin Methylene Blue (EMB) agar (Oxoid Ltd., Cambridge, UK) to obtain a pure colony. Colonies with a metallic green sheen on EMB (characteristic of *E. coli*) was later characterized microscopically using Gram's stain according to the method described by [46].

After isolation of the organism on the selective media, triple sugar iron (TSI) agar (Difco, MI, USA) was used for further characterization. Yellow slant, yellow butt, presence of gas bubbles, and absence of black precipitate in the butt were observed which indicates *E. coli* [47]. Then, the isolates were subjected to different biochemical tests according to [48] such as sugar fermentation test and indole production test, methyl-red, Voges-Proskauer, and citrate utilization (IMViC) test. Then, a bacterium that was confirmed as *E. coli* was subcultured onto Sorbitol MacConkey agar (Oxoid Ltd., Cambridge, UK) and was incubated at 37°C for 24 hours.

The nonsorbitol-fermenting (NSF) *E. coli* (colorless or pale colonies) was considered as *E. coli* O157 : H7 strains whereas pinkish-colored colonies (sorbitol-fermenters) were considered as non-O157 : H7 *E. coli* strains. The NSF isolates were again subjected to latex *E. coli* O157 : H7 agglutination test for confirmation.

#### 2.3.3. Latex Agglutination Test for *E. coli* O157 : H7

The rapid latex test kit is a rapid latex agglutination test that was intended for confirmatory identification of *E. coli* serogroup O157 [49]. The NSF isolates were inoculated onto nutrient agar for testing. Then, NSF and indole positive colonies were serotyped using Oxoid Dryspot *E. coli* O157 latex test kit. The Dryspot *E. coli* O157 latex test was demonstrated by agglutination of *Escherichia* strains possessing the O157 serogroup antigen. The test was conducted by just adding one drop of latex suspension and dispensing near the edge of the circle on the reaction card.

Then, a portion of a typical colony to be tested was emulsified using a loop in a drop of the sterile saline solution near the drop of test latex on the test card. After ensuring a smooth suspension of the bacteria and saline, the test latex was mixed with the suspension and spread to cover the reaction area over the loop. Then, the card was rocked in a circular motion for one minute and examined for agglutination by the naked eye. Agglutination of the test latex within one minute was considered as a positive result [49].

#### 2.3.4. Antimicrobial Susceptibility Testing for *E. coli* O157 : H7

The antimicrobial susceptibility of *Escherichia coli* O157 isolates was investigated using the disc diffusion method according to the guidelines for the Clinical and Laboratory Standards Institute [50]. The used antibiotics were amoxicillin-clavulanic acid (AMC 20 *μ*g), kanamycin (KAN 30 *μ*g), trimethoprim-sulfamethoxazole (SXT 25 *μ*g), chloramphenicol (CHL 30 *μ*g), ciprofloxacin (CPR 5 *μ*g), streptomycin (STR 10 *μ*g), nalidixic acid (NA 30 *μ*g), cefoxitin (CFX 30 *μ*g), tetracycline (TTC 30 *μ*g), and nitrofurantoin (NTR 50 *μ*g).

### 2.4. Data Management and Analysis

Data generated from laboratory investigations were recorded and coded using Microsoft Excel spreadsheet (Microsoft Corporation) and was analyzed using STATA version 14.0 for Windows (Stata Corp. College Station, TX, USA).

The prevalence of *E. coli* O157 : H7 isolated from the selected food of animal origin was calculated as the number of positive (confirmed) samples divided by the total number of samples investigated (processed) in the laboratory. Logistic regression and/or descriptive statistics such as frequency, percentage, and/or proportion were applied to compute the collected data from the selected foods of animal origin and antimicrobial susceptibility test results.

## 3. Results

### 3.1. Prevalence of *E. coli* O157 : H7 Isolated from Food of Animal Origin

In the present study, isolation and identification of *E. coli* O157 : H7 were performed by standard bacteriological methods and rapid latex agglutination kit, an immunological test, from the selected food of animal origin, and its prevalence was 2.1% (4/192). A higher prevalence of 4.3% was observed in Gobessa town compared to the other study areas but there was no significant association between the study settings and *E. coli* O157 isolated from food of animal origin as depicted in Table 1.

### 3.2. Chi-Square Analysis of Association of the Putative Risk Factors with *E. coli* O157 : H7 Occurrence in the Food of Animal Origin

A chi-square analysis revealed that catering establishments and cutting table (board) available were the only risk factors positively associated (*p* < 0.05) with *E. coli* O157 incidence among the putative risk factors contemplated during the study as illustrated in Table 2.

### 3.3. Multivariable Logistic Regression Analysis of Putative Risk Factors Associated with *E. coli* O157 : H7 Occurrence in Food of Animal Origin

A logistic regression analysis of the putative risk factors revealed that there were no putative risk factors associated with *E. coli* O157 : H7 occurrence in the food animal origin sampled from different catering establishments as elucidated in Table 3.

### 3.4. Occurrence of *E. coli* O157 : H7 in Food of Animal Origin and Its Antimicrobial Susceptibility Profile

All the four *E. coli* O157 : H7 isolates obtained in the current study were susceptible to ciprofloxacin, cefoxitin, amoxicillin-clavulanic acid, and trimethoprim-sulfamethoxazole whereas three of them were highly resistant to kanamycin, streptomycin, and nitrofurans. Nevertheless, there was no significant association between the antibiotics that the isolates were resistant to and *E. coli* O157 : H7 isolated from food of animal origin in the selected catering establishments of study settings.

### 3.5. Multiple Antimicrobial Resistance Profile of *E. coli* O157 : H7 Isolated from Food of Animal Origin

Of the four isolates obtained from “Kurt” (25%), “Dulet” (50%), and raw milk (25%), all of them were resistant to three or more antibiotics. A sole isolate obtained from “Kurt” was resistant to four antibiotics while an isolate obtained from “Dulet” was resistant to five antibiotics as depicted in Table 4.

## 4. Discussion

In the current study, 2.1 % (4/192) of an overall prevalence rate of *E. coli* O157 : H7 was isolated from the selected food of animal origin which closely agrees with the finding of [31] from beef carcass surface swabs at Haramaya University slaughterhouse (2.65%), [51] from beef carcass surface swabs at two commercial abattoirs in Samsun Province of Turkey (2%), and [3] from butcher shops in Central Ethiopia (1.7%). Contrarily, an overall prevalence of *E. coli* O157 : H7 observed in the present study was lower than [29, 52–54] who reported 4.2% (from Modjo and Bishoftu), 5.1% (from Modjo), 2.86% (from China), and 2.8% (from Iran), respectively. Such variations may be due to differences in the hygienic status of sources (catering establishments), type of samples, method of sampling, and culturing techniques. For, the presence of *E. coli* O157 : H7 in meats of ruminants can also be reasons as [55] indicated the equipment used for each operation in the abattoir, the clothing and hands of personnel and the physical facilities themselves can also be potential sources of microbial contaminations of *E. coli* in cattle meat slaughtered in abattoirs.

An isolate of *E. coli* O157 was detected in “Kurt” (3.1 %) (a traditional Ethiopian chopped meat, i.e., red meat that has a white fat that is consumed with or without “Berbere” (Ethiopian seasoning prepared from dry red chili peppers, garlic, and other spices) or “Mitmita” (bird's eye red pepper spiced with cardamom and salt). As per the researchers' best knowledge, *E. coli* O157 was isolated from “Kurt” collected from the consumers' dish samples for the first time in Ethiopia.

The raw “Dulet,” traditional Ethiopian minced tripe, liver, and lean beef fried in butter, onions, chili, cardamom, and pepper (often eaten for breakfast) is impossibly creamy and assertively spiced with “Mitmita,” Ethiopian blend of dried red chilies with cardamom, cloves, cinnamon, ginger, and cumin; it is among the delicious food of animal origin in our country. The liver in “Dulet” makes the mixture extraordinarily dense and creamy; the bits of tripe add textural contrast to another otherwise uniformly textured dish. In this study, the highest prevalence of 6.3 % (2/32) of *E. coli* O157 was isolated from “Dulet” among the rest of Ethiopian traditional minced meat considered in this study.

In the present study, the isolation rate of *E. coli* O157 : H7 from raw milk was 3.1% which is comparable to the prevalence report of 3.9% [56] from the raw milk analyzed in Germany, 3% [57] from milk samples tested in Austria, 2.9% [58] from traditionally marketed raw cow milk in Asossa town, Western Ethiopia, and 2.6% [59] from Egypt. However, the prevalence is far lower when compared to the reports of 33.5% [60] and 8.75% [61] from Malaysia, 12% [9] from Bishoftu town, Central Ethiopia, and 10.4% [62] from selected Woredas of Tigray, Ethiopia. This might be due to differences in animal management, milking systems, and milk handling practices among different countries [9, 58].

Regarding the risk factors associated with the prevalence of *E. coli* O157 : H7, estimates of the prevalence among catering establishments and cutting table (board) used vary considerably. The prevalence of *E. coli* O157 : H7 in foods of animal origin among catering establishments showed a significant difference (*p* < 0.05) in which *E. coli* O157 : H7 was not recovered in samples originated from the retail shop while the highest prevalence of *E. coli* O157 was obtained from hotel originated food items. This might be attributed to a lack of knowledge about good hygienic practices as none of the servants had taken formal training in food safety. It is a well-documented fact that poor personal hygiene is one of the most important sources of contamination for foods [63, 64].

In the current study, a significant difference of *E. coli* O157 : H7 was observed among the cutting table (board) available in the catering establishments to prepare minced meat in the study settings in which a prevalence rate of 16.2% was obtained in the catering establishments using separate cutting board, indicating the possible contamination of the wooden board by the carcass or vice versa. The isolation of *E. coli* O157 from the carcass in contact material, although the carcass itself was negative, may suggest the presence of other potential sources of contamination in catering establishments like cleaning water or inadequate cleaning and disinfection of the cutting boards leading to possible biofilm formation by the organisms on the wooden board [3]. Indeed, *E. coli* O157 has been isolated from water samples in Ethiopia [65] and biofilm formation of *E. coli* O157 in various food contact surfaces and tolerance to sanitizing reagents has been reported [66]. *E. coli* O157 contaminated cutting boards can be an important source of cross-contamination and may pose a significant public health risk.

Antimicrobial resistance of *E. coli* O157 : H7 isolates from animal and human sources has been reported in Ethiopia by [29]. In the present study, *E. coli* O157 : H7 showed resistance to five antimicrobials which varied from 100% to 50% except for ciprofloxacin, cefoxitin, amoxicillin-clavulanic acid, nalidixic acid, and trimethoprim-sulfamethoxazole to which 100% susceptibility was observed.

A 100% susceptibility of the four isolates to ciprofloxacin, cefoxitin, amoxicillin-clavulanic acid, and nalidixic acid, and trimethoprim-sulfamethoxazole, is consistent with the findings of [25] from poultry farms in Eastern Ethiopia, [3] from beef at butcher shops and restaurants in Central Ethiopia, [33] from beef among the raw meat considered in the study conducted at Addis Ababa, Ethiopia, and trimethoprim-sulfamethoxazole [33] from raw meat in Addis Ababa, and [3] from a study conducted on beef at butcher shops and restaurants in Central Ethiopia. Most of these antimicrobials are not commonly used in Ethiopia in the treatment of animals that served as a source of meat. Moreover, the susceptibility might have contributed to the effectiveness of these antimicrobials mostly against Gram-negative bacteria like those of the family of *Enterobacteriaceae* to which *E. coli* O157 : H7 belongs [33].

The highest resistance to streptomycin in this study is in agreement with [29] who reported antimicrobial resistance to *E. coli* O157 : H7 isolates from raw meat samples to some of the above-mentioned antimicrobials, especially to streptomycin. A significantly high level of resistance to this antimicrobial was probably an indication of their extensive usage in the veterinary sector for therapeutic and prophylactic purpose for both *E. coli* and other infections.

The presence of resistance against kanamycin is in agreement with the previous findings of [29] whose study showed that all the isolates were resistant to kanamycin; nonetheless, it disagrees with the report of [30, 31] in which all the *E. coli* isolates were found susceptible to Kanamycin.

Meanwhile, the current finding astonishingly revealed that 100% of *E. coli* O157 : H7 isolates were found to be resistant to nitrofurantoin which was inconsistent with the finding of [3] who reported 77.8% (9/525) of an isolate were susceptible to nitrofurantoin in butcher shops and restaurants in Central Ethiopia. The variation observed might be due to a high number of antibiotics used (*n* = 10) while compared to 7–8 antibiotics used in different studies conducted in Ethiopia, small sample size (*n* = 192) the sample types considered, and laboratory methods employed in the current study, and also the variation could be due to the expression of resistant gene code by the pathogen which is associated with emerging and reemerging aspects of the isolates with regard to the different agroecology [67].

The four *E. coli* O157 : H7 isolates in the present study exhibited resistance to at least three or more of the ten antimicrobial agents used. Compared to this finding, [9] reported 100% resistance of all the isolates to more than two drugs from milk in Bishoftu town and [71] also reported 100% resistance rate of the isolates to two or more drugs, in an abattoir-based study conducted in eastern Ethiopia and this was in agreement with the current finding. Likewise, in this study, a higher rate of multidrug-resistance was observed for three drugs (50%) followed by four (25%) and five (25%) antibiotics. In contrast to this, the resistance of 28.6% to three and 14.3% to four drugs was reported by [9]. Meanwhile, in this study, resistance to five antimicrobials was recorded in one (25%) isolate from “Dulet.” The current finding was lower when compared with the reports of [67] in Nigeria who have reported a 52.6% resistance rate to seven antimicrobials, but higher than the report of [68] who reported 7.41%, 18.52%, and 11.11% resistance rate to five, six, and seven antimicrobials, respectively, in Central Ethiopia and [67] 15.8% resistance rate to eight drugs in Nigeria. Therefore, the development of antibiotic resistance among bacteria such as *E. coli* poses an important public health concern.

The variation in the development of multidrug-resistance for the bacteria may be due to the variation in dose, route of administration, regimen, and continuous and indiscriminate use of antimicrobials for treatment and feed additive in various study areas and level of awareness and geographic location for studies abroad. The effectiveness of treatments and the ability to control infectious diseases in both animals and humans may be severely hampered due to rapid development of multidrug resistance [69].

## 5. Conclusion

The most significant food-borne pathogens that have gained increased attention in recent years is *E. coli* O157 : H7. In the current study, *E. coli* O157 : H7 isolates were recovered from the food of animal origin, namely, raw/minced meat (“Kurt” and “Dulet”) and raw milk, in the selected areas of Arsi Zone. Catering establishments and cutting table (board) were the only risk factors that positively associated with *E. coli* O157 occurrence among the putative risk factors contemplated in the catering establishments. Three-fourths (3/4) of the isolates recovered from the stipulated foods of animal origin were highly resistant to kanamycin, streptomycin, and nitrofurantoin whereas all of the isolates obtained from this study displayed multidrug resistance, 3 to 5 antibiotics, amid kanamycin, streptomycin, nitrofurantoin, tetracycline, and chloramphenicol antibiotics. Consequently, the occurrence of *E. coli* O157 : H7 in Ethiopian traditional minced meat (“Kurt” and “Dulet”) and raw milk and the existence of multidrug-resistant isolates revealed that there is a risk for public health and food safety as well as animal production in the study setting. Thus, this finding calls for urgent attention toward appropriate controls and good hygienic practices in different catering establishments dealing with consuming raw/undercooked animal origin food items.

## Figures and Tables

**Table 1:** Prevalence of *E. coli* O157 : H7 isolated from selected food of animal origin in the study settings.

Variable category	Samples tested	Result of tested samples		*χ*2 (*p* value)
		Negative sample *N* (%)	Positive sample *N* (%)	
Study settings				2.435 (0.706)
Dera	37	36 (97.3)	1 (2.7)	
Iteya	31	31 (100)	0	
Asella	64	62 (96.9)	2 (3.1)	
Bekoji	37	37 (100)	0	
Gobessa	23	22 (95.7)	1 (4.3)	
**Total**	**192**	**188 (97.9)**	**4 (2.1)**	

*χ*2, Pearson's chi-square; *N*, number of samples.

**Table 2:** Chi-square analysis of the association of the putative risk factors with *E. coli* O157 : H7 occurrence in the food of animal origin in the study settings of Arsi Zone.

Variables category	Samples tested	Positive sample *N* (%)	*χ* ^2^ (*p* value)
Sample type			5.106 (0.717)
Kitfo	32	0	
Kurt	32	1 (3.1)	
Dulet	32	2 (6.3)	
Egg sandwich	32	0	
Raw milk	32	1 (3.1)	
Cream cake	32	0	

Catering establishments			11.082 (0.036^*∗*^)
Hotel	14	2 (14.3)	
Restaurant	82	1 (1.2)	
Cafeteria	87	1 (1.1)	
Retail shop	9	0	

Protective clothing			2.107 (0.147)
Used	152	2 (1.3)	
Not used	40	2 (5)	

Source of contamination			0.065 (0.799)
Unclean cutting board#	3	0	
Handling with unclean equipment and hands	189	4 (2.1)	

Manner of hand washing			0.272 (0.602)
Rinsing with water only	72	1 (1.4)	
Using detergents and water	120	3 (2.5)	

Money handling			0.449 (0.990)
Butcher with bare hand#	11	0	
Cashier	8	0	
Service woman/man	173	4 (2.3)	

Cutting table (board) available			13.350 (0.018^∗^)
Single for minced meat	84	1 (1.2)	
Separate for minced meat	12	2 (16.7)	
Not available	96	1 (1.0)	

Origin of the sample			2.435 (0.695)
Dera	37	1 (2.7)	
Eteya	31	0	
Asella	64	2 (3.1)	
Bekoji	37	0	
Gobessa	23	1 (4.3)	

#For minced meat; ^∗^statistically significant.

**Table 3:** Multivariable logistic regression analysis of putative risk factors associated with *E. coli* O157 : H7 occurrence in animal origin food items in the study sites.

Variables category	Samples tested	Positive sample N (%)	Odds ratio		*p* value
			COR (95%CI)	AOR (95%CI)	
Catering establishments					0.159
Hotel	14	2 (14.3)	R	R	
Restaurant	82	1 (1.2)	2.692E8 (0.000, -)	3.414E8 (0.000, -)	
Cafeteria	87	1 (1.1)	1.994E7 (0.000, -)	1.418E8 (0.000, -)	
Retail shop	9	0	1.878E7 (0.000, -)	1.878E7 (0.000, -)	

Cutting table (board) availability					0.441
Single for minced meat	84	1 (1.2)	R	R	
Separate for minced meat	12	2 (16.7)	1.145 (0.070, 18.587)	0.131 (0.001, 23.138)	
Not available	96	1 (1.0)	19 (1.580, 228.551)	-	

AOR, adjusted odds ratio; CI, confidence interval; COR, crude odds ratio; R, reference.

**Table 4:** Multiple antimicrobial resistance profile of *E. coli* O157 isolated from food of animal origin in the selected catering establishments of the study sites.

Number of antimicrobial resistances	Antimicrobial resistance patterns (#)	Number of isolates (%) (*n* = 4)
Three	KAN, STR, NTR (1)	2 (50)
	STR, NTR, TTC (1)	

Four	KAN, STR, NTR, TTC (1)	1 (25)
Five	KAN, STR, NTR, TTC, CHL (1)	1 (25)
Total		4 (100)

KAN, kanamycin; STR, streptomycin; NTR, nitrofurantoin; TTC, tetracycline; CHL, chloramphenicol; #, number of isolate (s) resistant to a group of antibiotics.

## Data Availability

The data used to support the findings of this study are available from the corresponding author upon request.
